# Identification of new candidates regulating autophagy-dependent midgut degradation in *Drosophila melanogaster*

**DOI:** 10.1038/s41420-025-02474-0

**Published:** 2025-04-16

**Authors:** Ruchi Umargamwala, Shannon Nicolson, Jantina Manning, Julian M. Carosi, Sharad Kumar, Donna Denton

**Affiliations:** 1 Centre for Cancer Biology, University of South Australia, Adelaide, Australia; 2 South Australian Health and Medical Research Institute, Adelaide, Australia; 3 School of Biological Sciences, The University of Adelaide, Adelaide, Australia; 4 Faculty of Health and Medical Sciences, The University of Adelaide, Adelaide, Australia

**Keywords:** Autophagy, Cell growth

## Abstract

Autophagy-dependent cell death (ADCD) is a context-specific form of programmed cell death that plays an important role in development and homeostasis. During *Drosophila* metamorphosis, hormonal cues modulate growth and other signalling cascades which results in autophagy-dependent degradation of the obsolete larval midgut. While this process does not require caspase activity or apoptotic machinery, several canonical autophagy-related proteins are also dispensable, suggesting additional regulators may be involved in effectively eliminating the larval midgut. Ubiquitination, a process that attaches one or more ubiquitin moieties to a substrate through sequential reactions involving a cascade of enzymes, plays a critical role in autophagy. As the specific role(s) of ubiquitination in ADCD has not been explored, we previously performed a RNAi-mediated knockdown screen of over 250 ubiquitin machinery genes in GFP-labelled *Drosophila* larval midguts and identified 18 candidate regulators of midgut degradation. In this work, we screened candidate genes for a role in autophagy-dependent midgut degradation by analysing mosaic clones and genetic interactions with *Atg1*. Validation and further studies into the ubiquitin conjugating enzyme, Effete (Eff), and two ubiquitin ligases, Cullin-4 (Cul4) and Supernumerary limbs (Slmb), demonstrated interplay between ubiquitination and the autophagy machinery in coordinating autophagy-dependent midgut degradation.

## Introduction

Programmed cell death is essential for organismal development and homeostasis by coordinating morphogenesis, organ formation and removal of aberrant cells [1]. Although caspase-dependent apoptosis is commonly utilised for developmental programmed cell death, ADCD occurs under specific contexts, relying exclusively on autophagy machinery to mediate lysosomal degradation of bulk substrates [2, 3]. In *Drosophila*, developmental programmed cell death is spatiotemporally-regulated by the steroid hormone, 20-hydroxyecdysone (20E, ecdysone), which binds to nuclear hormone receptors to regulate various signalling cascades [4]. During metamorphosis, transition of the *Drosophila* larva to an adult occurs in response to pulses of ecdysone release [5]. Specifically, the late larval ecdysone pulse triggers autophagy-dependent degradation of the obsolete midgut, resulting in rapid destruction of gastric caeca appendages and the proventriculus [6]. Ecdysone-controlled downregulation of PI3K-dependent growth signalling, and the morphogen, decapentaplegic (Dpp), are crucial for initiating ADCD in the larval midgut whereas caspase activity is dispensable [7–9]. However, not all members of the canonical autophagy machinery, such as *Atg7*, are required for effective midgut degradation, suggesting the involvement of other regulatory mechanisms in this process [10–12].

Post-translational modifications are required to tightly control autophagy. Ubiquitination of core autophagy-related proteins by various ubiquitin ligases (E3s) can affect protein stability and alter signalling properties, in turn fine-tuning autophagy at all stages [13]. For example, the E3 ligase, Tumour necrosis factor receptor (TNFR)-associated factor 6 (TRAF6), promotes ubiquitin-dependent ULK1 stability [14]. In *Drosophila*, the E1 enzyme, Uba1, is involved in the clearance of cytoplasmic material in midgut cells in an Atg8a-dependent manner [15]. More recently, the *Drosophila* Really Interesting New Gene (RING) domain-containing E3 ligase, detour, was identified as a regulator of autophagosome biogenesis and facilitator of autophagosome-lysosome fusion by interacting with homotypic fusion and vacuole protein sorting (HOPS) complex subunits [16]. With emerging evidence of the importance of ubiquitination in autophagy-dependent midgut degradation, it is unknown whether there are additional uncharacterised members of the ubiquitin system that are involved in this process.

In our previous work, we performed RNAi-mediated knockdown of >250 components of the ubiquitination machinery in *Drosophila* larval midguts [17]. This identified 18 enzymes involved in ubiquitination that caused defects in midgut degradation. These were validated by a mosaic clone secondary screen for the autophagosome marker, Atg8a, and their interaction with the autophagy initiation complex gene, *Atg1*, was investigated in the developing adult eye. Further investigations into the knockdown effect of the E2 enzyme, Effete (Eff), and E3 ligases, Cullin-4 (Cul4) and Supernumerary limbs (Slmb), in the larval midgut revealed drastic midgut remodelling and significant alterations to autophagosomal and lysosomal compartments during metamorphosis. Overall, this study implicates previously uncharacterised roles of ubiquitination enzymes in *Drosophila* autophagy-dependent midgut degradation and expands on our understanding of how ADCD is regulated in developmental contexts.

## Results

### A genetic screen for regulators of autophagy-dependent midgut degradation

To identify new regulators of autophagy-dependent midgut degradation, we previously performed RNAi-mediated knockdown of >250 components of the ubiquitination machinery in *Drosophila* larval midguts [17]. These genes encoded enzymes that functioned in ubiquitin activation (E1), ubiquitin conjugation (E2) and ubiquitin ligation (E3) [17]. Candidate genes were knocked down in the midgut using *mex-GAL4* > *UAS-EGFP* flies crossed to *UAS-RNAi* lines encoding ubiquitination enzymes and progeny were examined for morphological differences in midgut remodelling using EGFP fluorescence at 0 h relative to puparium formation (h RPF) [17, 18]. The expression of *UAS-RNAi* lines targeting 18 components of the ubiquitination machinery resulted in phenotypes that included developmental arrest, alterations to EGFP patterning, inhibition of midgut degradation and premature midgut degradation (Supplementary Table 2) [17].

To assess how these genes altered developmental autophagy during midgut degradation following RNAi-mediated knockdown, we generated mosaic clones in larval midgut cells in which cells expressing *UAS-RNAi* transgenes labelled with GFP were compared to adjacent non-GFP control cells. Autophagy was monitored using Atg8a tagged with mCherry (mCherry-Atg8a), and the number and size of Atg8a-positive vesicles was quantified at the onset of autophagy induction at -4 h RPF [19]. During midgut degradation, downregulation of growth signalling results in robust levels of autophagy, leading to cell size reduction that is prevented upon autophagy inhibition [8]. Therefore, the sizes of cell clones were also measured as a readout of cell size reduction upon *UAS-RNAi* transgene expression. Subsequently, the 18 identified ubiquitination enzymes have been grouped according to the most significant phenotype exhibited upon knockdown: increased the number of Atg8a-positive vesicles, decreased the number of Atg8a-positive vesicles, and increased the size of Atg8a-positive vesicles.

### Genes that cause an increase in the number of Atg8a-positive vesicles upon knockdown

**Fig. 1:**
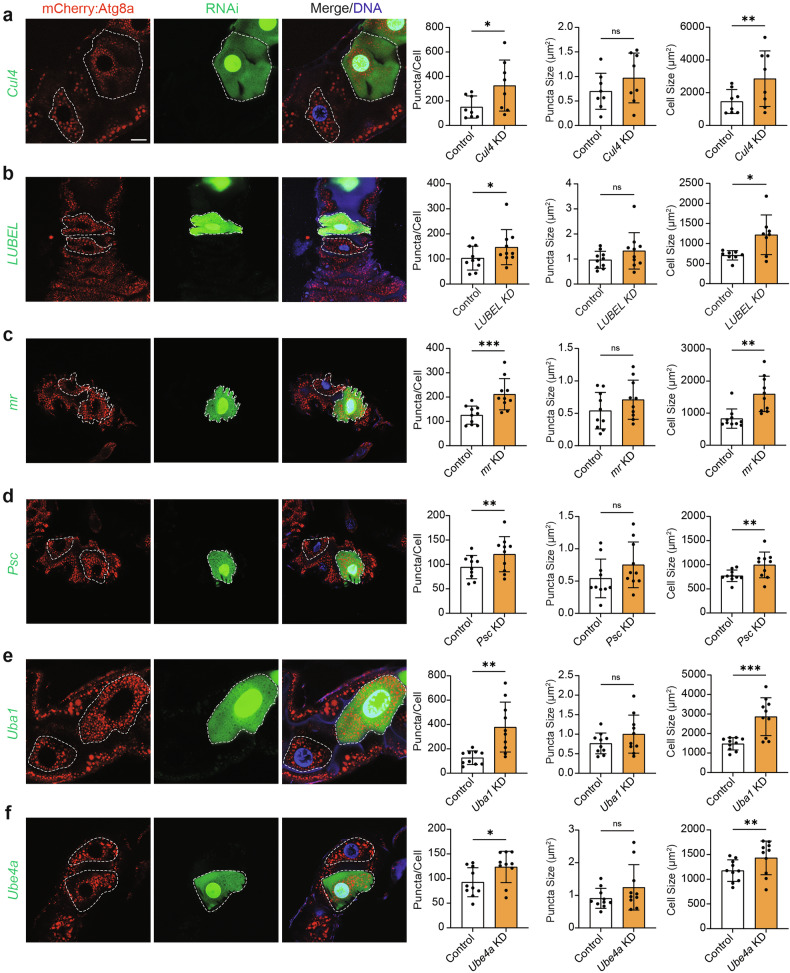
Mosaic clone mutants that increase the number of Atg8a puncta. **a** *Cul4* KD (*hsFLP; pmCherry-Atg8a/+; Act* *>* *CD2* *>* *GAL4, UAS-nlsGFP/UAS-Cul4i*), (**b**) *LUBEL* KD (*hsFLP; pmCherry-Atg8a/+; Act* *>* *CD2* *>* *GAL4, UAS-nlsGFP/UAS-LUBELi*), (**c**) *mr* KD (*hsFLP; pmCherry-Atg8a/UAS-mri; Act* *>* *CD2* *>* *GAL4, UAS-nlsGFP/+*), (**d**) *Psc* KD (*hsFLP; pmCherry-Atg8a/UAS-Psci; Act* *>* *CD2* *>* *GAL4, UAS-nlsGFP/+*), (**e**) *Uba1* KD (*hsFLP; pmCherry-Atg8a/+; Act* *>* *CD2* *>* *GAL4, UAS-nlsGFP/UAS-Uba1i*) and (**f**) *Ube4a* KD (*hsFLP; pmCherry-Atg8a/+; Act* *>* *CD2* *>* *GAL4, UAS-nlsGFP/UAS-Ube4ai*) in GFP-labelled midgut cells compared to non-GFP control cells (white dashed lines indicate cell boundaries) at -4 h RPF. Quantitation of Atg8a puncta per cell represented as puncta/cell ± SD (paired *t*-test, *n* ≥ 8). Quantitation of Atg8a puncta size represented as µm^2^ ± SD (paired *t*-test, *n* ≥ 8). Quantitation of cell size represented as µm^2^ ± SD (paired *t*-test, *n* ≥ 8). Scale bar = 20 µm.

Upon depletion of the E1 enzyme, *Uba1*, and E3 enzymes, *Cul4*, *LUBEL*, *mr*, and *Psc*, and the E3/E4 enzyme, *Ube4a*, we observed a significant increase in the number of Atg8a-positive vesicles at -4 h RPF, although no differences in Atg8a-positive vesicle size were found (Fig. 1a–f). These clones were also significantly larger than neighbouring control cells, suggesting a relationship between cell size and increased Atg8a-positive vesicle abundance.

**Fig. 2:**
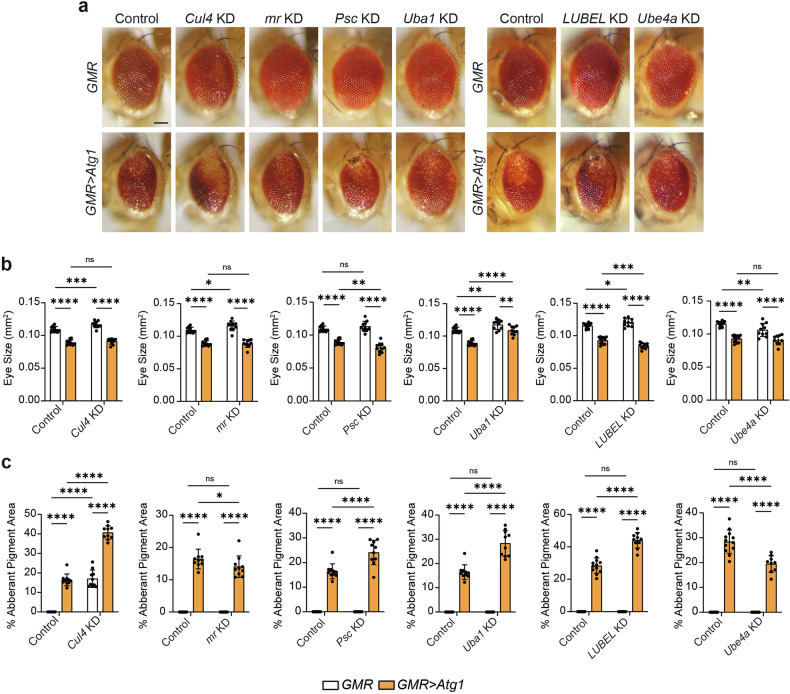
Eye morphology phenotypes for genes that increase Atg8a-positive vesicles upon knockdown in clones. **a** Representative eye phenotypes for RNAi lines crossed with *GMR-GAL4* (*GMR-GAL4/* + *; UAS-Cul4i/+*, *GMR-GAL4/UAS-mri*, *GMR-GAL4/UAS-Psci*, *GMR-GAL4/* + *; UAS-Uba1i/+*, *GMR-GAL4/* + *; UAS-LUBELi/+* and *GMR-GAL4/* + *; UAS-Ube4ai/+*) and *GMR>Atg1* (*GMR-GAL4; GMR>Atg1/UAS-Cul4i*, *GMR-GAL4/UAS-mri; GMR>Atg1/+*, *GMR-GAL4/UAS-Psci; GMR>Atg1/+*, *GMR-GAL4/* + *; GMR>Atg1/UAS-Uba1i*, *GMR-GAL4/* + *; GMR>Atg1/UAS-LUBELi*, *GMR-GAL4/* + *; GMR>Atg1/UAS-Ube4ai*) compared to controls (*GMR-GAL4/w*^*1118*^ and *GMR-GAL4/w*^*1118*^*; GMR>Atg1/w*^*1118*^). Separate *GMR-GAL4* and *GMR>Atg1* controls are shown due to differences in experimental timing. **b** Quantitation of eye size represented as mm^2^ ± SD (Two-way ANOVA with Uncorrected Fisher’s LSD test, *n* ≥ 10). **c** Quantitation of aberrant pigment area represented as % Aberrant Pigment Area ± SD (Two-way ANOVA with Uncorrected Fisher’s LSD test, *n* ≥ 10). Scale bar = 125 μm.

The autophagy initiation complex gene, *Atg1* (ULK1 in mammals), is critical for proper contraction of gastric caeca and midgut condensation during autophagy-dependent midgut degradation [8, 10]. In the adult *Drosophila* eye, overexpression of *Atg1* induces hyperautophagy that results in caspase-dependent cell death, leading to disruption of eye architecture, de-pigmentation of eye colour and reduction of eye size [20]. Therefore, to determine if candidate genes genetically interacted with *Atg1*, we utilised the eye-specific driver gene, *GMR-GAL4*, to perform RNAi-mediated knockdown of candidate genes under basal (*GMR* only) and *Atg1* overexpression conditions (*GMR>Atg1*). Depletion of *Cul4*, *mr*, *Uba1* and *LUBEL* caused a significant increase in eye size, whereas *Ube4a* KD decreased eye size, with *GMR* alone compared to GMR controls (Fig. 2a, b). Knockdown of all genes resulted in significant reduction of eye size with *Atg1* compared to their knockdown with *GMR* alone (Fig. 2a, b). *Psc* KD and *LUBEL* KD eyes with *Atg1* overexpression were significantly smaller than *Atg1* control eyes, whereas *Uba1* KD eyes with *Atg1* overexpression were significantly larger compared to *Atg1* control eyes (Fig. 2b). Notably, while *mr* KD and *Ube4a* KD reduced the area of aberrant tissue, *Cul4* KD, *Psc* KD, *Uba1* KD and *LUBEL* KD caused disruption to ommatidial arraying and led to larger areas of pigment loss when *Atg1* was overexpressed (Fig. 2c).

Overall, this reveals a genetic interaction between genes that regulate Atg8a-positive vesicle number and cell size in mosaic clones and *Atg1*, providing greater evidence for their potential functional roles in regulating midgut ADCD.

### Genes that cause a decrease in the number of Atg8a-positive vesicles upon knockdown

**Fig. 3:**
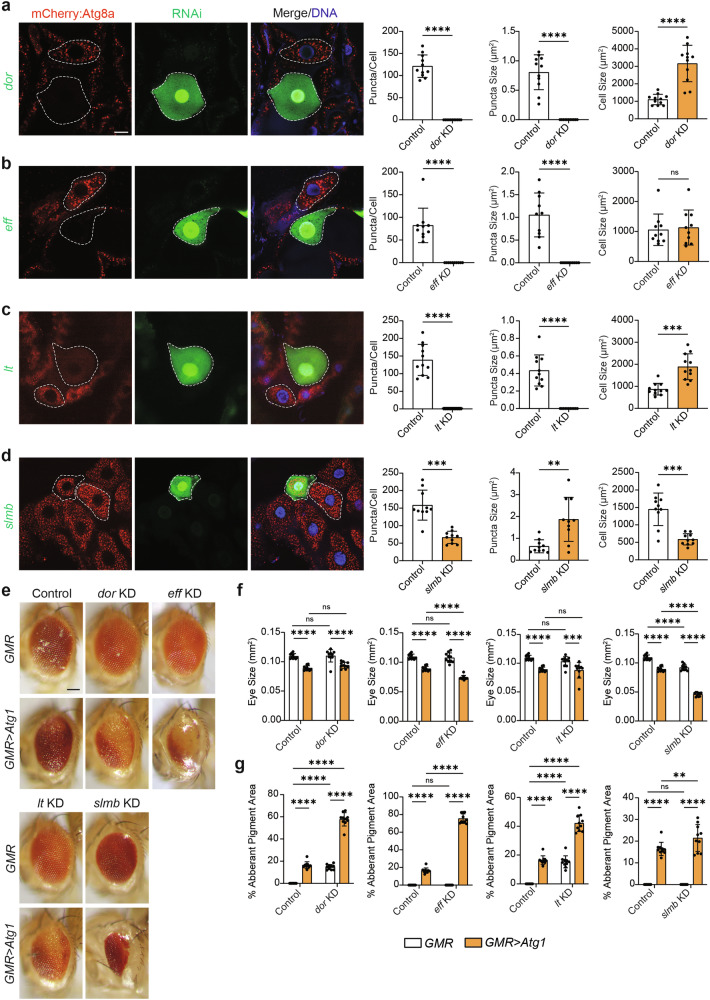
Mosaic clone knockdowns that decrease the number of Atg8a-positive vesicles and their representative eye morphologies. **a** *dor* KD (*hsFLP; pmCherry-Atg8a/+; Act* > *CD2* > *GAL4, UAS-nlsGFP/UAS-dori*), (**b**) *eff* KD (*hsFLP; pmCherry-Atg8a/+; Act* > *CD2* > *GAL4, UAS-nlsGFP/UAS-eff*^*35431*^*i*), (**c**) *lt* KD (*hsFLP; pmCherry-Atg8a/UAS-lti; Act* > *CD2* > *GAL4, UAS-nlsGFP/+*) and (**d**) *slmb* KD (*hsFLP; pmCherry-Atg8a/+; Act* > *CD2* > *GAL4, UAS-nlsGFP/UAS-slmbi*) in GFP-labelled midgut cells compared to non-GFP control cells (white dashed lines indicate cell boundaries) at -4 h RPF. Quantitation of Atg8a puncta per cell represented as puncta/cell ± SD (paired *t*-test, *n* ≥ 10). Quantitation of Atg8a puncta size represented as µm^2^ ± SD (paired *t*-test, *n* ≥ 10). Quantitation of cell size represented as µm^2^ ± SD (paired *t*-test, *n* ≥ 10). **e** Representative eye phenotypes for RNAi lines crossed with *GMR-GAL4* (*GMR-GAL4/* + *; UAS-dori/+*, *GMR-GAL4/* + *; UAS-eff*^*35431*^*i/+*, *GMR-GAL4/UAS-lti*, *GMR-GAL4/* + *; UAS-slmbi/+*) and *GMR>Atg1* (*GMR-GAL4/* + *; GMR>Atg1/UAS-dori*, *GMR-GAL4/* + *; GMR>Atg1/UAS-eff*^*R2*^*i*, *GMR-GAL4/UAS-lti; GMR>Atg1/+*, *GMR-GAL4/* + *; GMR>Atg1/UAS-slmbi*) compared to controls (*GMR-GAL4/w*^*1118*^ and *GMR-GAL4/w*^*1118*^*; GMR>Atg1/ w*^*1118*^). **f** Quantitation of eye size represented as mm^2^ ± SD (Two-way ANOVA with Uncorrected Fisher’s LSD test, *n* = 10). **g** Quantitation of aberrant pigment area represented as % Aberrant Pigment Area ± SD (Two-way ANOVA with Uncorrected Fisher’s LSD test, *n* = 10). Scale bar = 20 μm (**a**–**d**) and 125 μm (**e**).

Several genetic knockdowns resulted in fewer Atg8a-positive vesicles in mosaic clones. Depletion of *dor*, *eff* and *lt* abolished Atg8a-positive vesicles (Fig. 3a–c). However, only *dor* KD and *lt* KD prevented midgut cell size reduction which has previously been shown to occur as a result of defective downregulation of growth signalling prior to midgut degradation (Fig. 3a, c) [16]. *slmb* KD clones showed a decrease in the number of Atg8a-positive vesicles that were also significantly larger in size (Fig. 3d). This was correlated to a decrease in cell size when compared to non-GFP control cells, possibly due to a faster rate of cell removal (Fig. 3d). Subsequently, these genes were also screened for their interaction with *Atg1* in the developing adult eye (Fig. 3e). Strikingly, loss of *slmb* resulted in a significant reduction in eye size and increased eye sphericity with *GMR* alone compared to controls (Fig. 3f). *dor*, *eff*, *lt* and *slmb* depletions reduced eye size when *Atg1* was overexpressed compared to their knockdowns with *GMR* alone (Fig. 3f). Only *eff* KD and *slmb* KD exacerbated the *Atg1* small eye phenotype compared to controls overexpressing *Atg1* (Fig. 3f). *dor* KD and *lt* KD caused pigment loss with *GMR* alone which was further driven by *Atg1* overexpression compared to *Atg1* controls (Fig. 3g). Loss of *eff* and *slmb* also led to significant increases in the percentage of aberrant pigment, total degeneration of ommatidia and smoothening of the eye surface (Fig. 3g).

Overall, our screens suggest that this subset of genes may be required for maintaining the number of Atg8a-positive vesicles during autophagy-dependent midgut degradation by interacting with *Atg1*.

### Genes that increase the size of Atg8a-positive vesicles upon knockdown

**Fig. 4:**
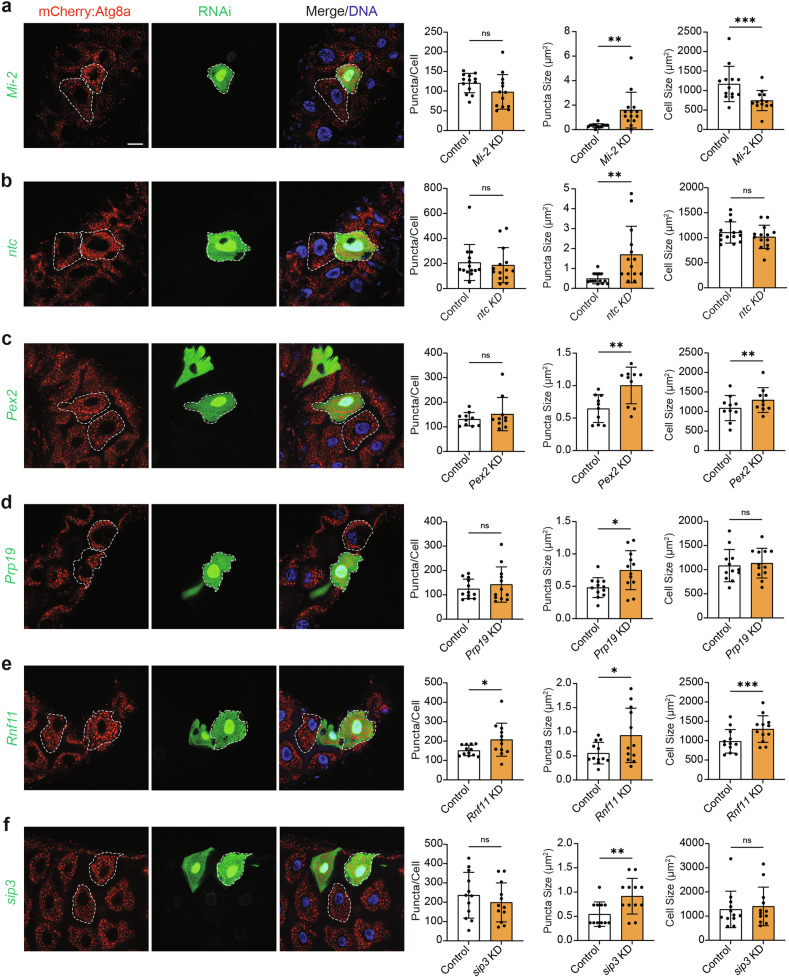
Mosaic clone mutants that cause an increase in Atg8a-positive vesicle size. **a** *Mi-2* KD (*hsFLP; pmCherry-Atg8a/+; Act* *>* *CD2* *>* *GAL4,* *UAS-nlsGFP/UAS-Mi-2i*), (**b**) *ntc* KD (*hsFLP; pmCherry-Atg8a/UAS-ntci; Act* *>* *CD2* *>* *GAL4, UAS-nlsGFP/+*), (**c**) *Pex2* KD (*hsFLP; pmCherry-Atg8a*/*+*; *Act* > *CD2* > *GAL4*, *UAS-nlsGFP*/*UAS-Pex2i*), (**d**) *Prp19* KD (*hsFLP; pmCherry-Atg8a/UAS-Prp19i; Act* *>* *CD2* *>* *GAL4, UAS-nlsGFP/+*), (**e**) *Rnf11* KD (*hsFLP; pmCherry-Atg8a/UAS-Rnf11i; Act* *>* *CD2* *>* *GAL4, UAS-nlsGFP/+*) and (**f**) *sip3* KD (*hsFLP; pmCherry-Atg8a/UAS-sip3; Act* *>* *CD2* *>* *GAL4, UAS-nlsGFP/+*) in GFP-labelled midgut cells compared to non-GFP control cells (white dashed lines indicate cell boundaries) at -4 h RPF. Quantitation of Atg8a puncta per cell represented as puncta/cell ± SD (paired *t*-test, *n* ≥ 10). Quantitation of Atg8a puncta size represented as µm^2^ ± SD (paired *t*-test, *n* ≥ 10). Quantitation of cell size represented as µm^2^ ± SD (paired *t*-test, *n* ≥ 10). Scale bar = 20 µm.

Our final subset of genes was classified based on their significant effect on the size of Atg8a-positive vesicles upon knockdown, which can differ depending on the type and amount of cytoplasmic material accumulated within Atg8a-positive vesicles [21]. *Mi-2* KD mosaic clones had increased Atg8a-positive vesicle size and decreased cell size, whereas a cell size difference was not observed for *ntc* KD mosaic clones where Atg8a-positive vesicles were significantly larger than in control cells (Fig. 4a, b). *Pex2* KD mosaic clones also contained large Atg8a-positive vesicles but this was correlated to increased size of clones compared to controls (Fig. 4c). In contrast, the large Atg8a-positive vesicles in *Prp19* KD clones did not affect cell size (Fig. 4d). *Rnf11* KD mosaic clones also showed a correlation between the increased number of large Atg8a-positive vesicles and increased cell size, however, *sip3* KD clones with larger Atg8a-positive vesicles did not show differences in mosaic clone cell size (Fig. 4e, f). This suggests that during midgut degradation, the contents of Atg8a-positive vesicles, which are known to influence their size, may play a key role in driving midgut cell size reduction and removal.

**Fig. 5:**
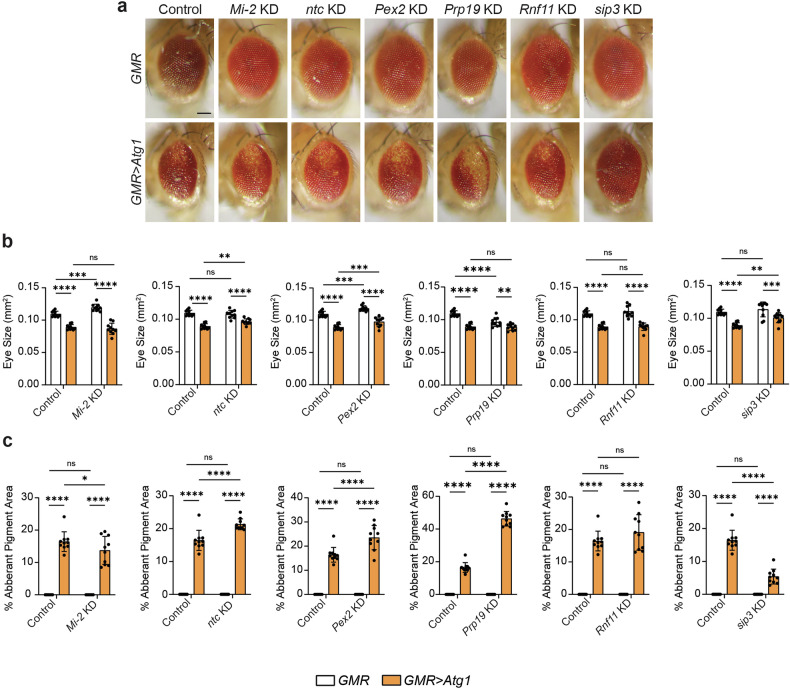
Eye morphology phenotypes for genes that increase Atg8a-positive vesicle upon knockdown in clones. **a** Representative eye phenotypes for RNAi lines crossed with *GMR-GAL4* (*GMR-GAL4/* + *; UAS-Mi-2i/+, GMR-GAL4/UAS-ntci, GMR-GAL4/* + *; UAS-Pex2i/+, GMR-GAL4/UAS-Prp19i, GMR-GAL4/UAS-Rnf11i* and *GMR-GAL4/UAS-sip3i*) and *GMR>Atg1* (*GMR-GAL4/* + *; GMR>Atg1/UAS-Mi-2i, GMR-GAL4/UAS-ntci; GMR>Atg1/+; GMR-GAL4/* + *; GMR>Atg1/UAS-Pex2i, GMR-GAL4/UAS-Prp19i; GMR>Atg1/+, GMR-GAL4/UAS-Rnf11i; GMR>Atg1/+* and *GMR-GAL4/UAS-sip3i; GMR>Atg1/+*) compared to controls (*GMR-GAL4/w*^*1118*^ and *GMR-GAL4/w*^*1118*^*; GMR>Atg1/ w*^*1118*^). **b** Quantitation of eye size represented as mm^2^ ± SD (Two-way ANOVA with Uncorrected Fisher’s LSD test, *n* = 10). **c** Quantitation of aberrant pigment area represented as % Aberrant Pigment Area ± SD (Two-way ANOVA with Uncorrected Fisher’s LSD test, *n* = 10). Scale bar = 125 μm.

Next, we determined if these genes had an interaction with *Atg1* (Fig. 5a). With *GMR* alone, *Mi-2* KD and *Pex2* KD resulted in larger eyes, whereas *Prp19* KD caused a reduction in eye area, compared to controls (Fig. 5b). All genes significantly reduced eye size upon knockdown with *Atg1* overexpression when compared to knockdown with *GMR* alone, however, *ntc* KD, *Pex2* KD, and *sip3* KD eyes were significantly larger compared to *Atg1* controls (Fig. 5b). *Mi-2* KD and *sip3* KD decreased the percentage of aberrant pigment, whereas *ntc* KD, *Pex2* KD and *Prp19* KD increased the severity of pigment loss (Fig. 5c). Therefore, it is possible that the interaction between these genes and *Atg1* may influence Atg8a-positive vesicle size to regulate timely removal of the larval midgut by ADCD.

### Effete

**Fig. 6:**
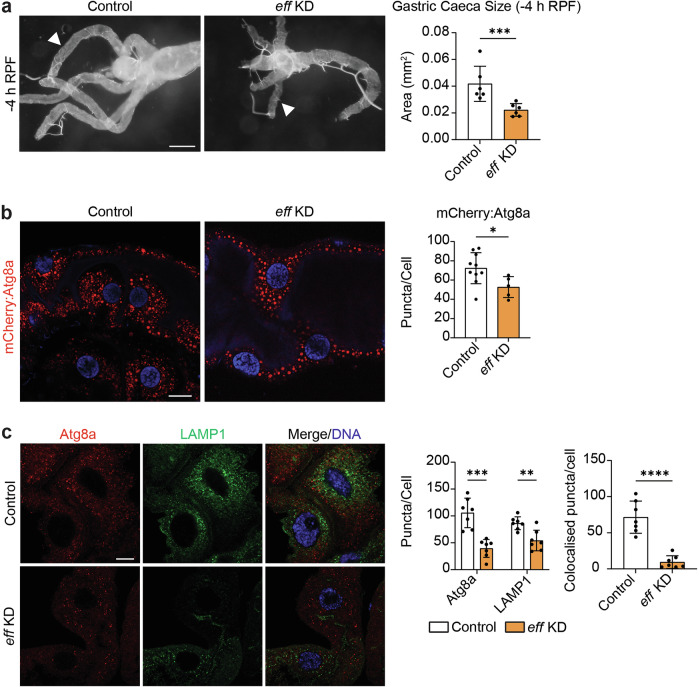
*eff* KD accelerates *Drosophila* midgut remodelling. **a** *eff* KD (*Mex-GAL4*/*+; pmCherry-Atg8a/UAS- eff*^*35431*^*i*) midguts have shorter gastric caeca compared to controls (*Mex-GAL4*/*w*^*1118*^*; pmCherry-Atg8a/+*). Quantitation of gastric caeca length at -4 h RPF. Data presented as area ± SD (unpaired *t*-test, *n* = 6). **b** *eff* KD (*Mex-GAL4/+; pmCherry-Atg8a/UAS- eff*^*35431*^*i*) midguts have significantly fewer Atg8a-positive vesicles (red) compared to controls (*Mex-GAL4*/*w*^*1118*^*; pmCherry-Atg8a/+*) at -4 h RPF. DNA stained by Hoechst (Blue). Quantitation of the number of Atg8a-positive vesicles represented as puncta/cell ± SD (unpaired t-test, *n* ≥ 5). **c** Immunostain for Atg8a (red) and LAMP1 (green) revealed fewer Atg8a-positive vesicles and LAMP1-positive vesicles in *eff* KD (*Mex-GAL4* > *UAS-GFP-LAMP1;* UAS-*eff*^*35431*^*i/+*) midguts compared to controls (*Mex-GAL4* > *UAS-GFP-LAMP1*/*w*^*1118*^). DNA stained by Hoechst (Blue). Quantitation of Atg8a-positive and LAMP1-positive puncta represented as puncta/cell ± SD (unpaired *t*-test, *n* = 7). Quantitation of overlap between Atg8a-positive compartment and LAMP1-positive compartment represented as colocalised puncta/cell ± SD (unpaired *t*-test, *n* = 7). Scale bars = 125 µm (**a**), 20 µm (**b**) and 10 µm (**c**).

The E2 enzyme, Eff, has been shown to function at the interface between autophagy and ubiquitin machinery [22, 23]. As the only E2 enzyme that caused complete depletion of Atg8a-positive vesicles upon knockdown in midgut clones, we investigated the role of Eff in midgut degradation utilising the midgut-specific driver, *mex-GAL4*, to perform a midgut-wide RNAi knockdown of *eff*. Morphological analyses indicated *eff* KD midguts were extensively remodelled and had smaller gastric caeca compared to controls (Fig. 6a). This is contrary to *eff* KD mosaic clone midgut cells that showed no differences in cell size compared to neighbouring control cells, suggesting a potential non-cell-autonomous role for Eff in regulating midgut degradation.

We then examined the number of Atg8a-positive vesicles in -4 h RPF midguts depleted of *eff*. *eff* KD midguts contained fewer Atg8a-positive vesicles when compared to control midguts which was consistent with their mosaic clone phenotype (Fig. 6b). To test interactions between autophagosomes and lysosomes in *eff* KD midgut cells, we performed immunostaining for Atg8a and the lysosomal membrane marker, GFP-LAMP1, in control and *eff* KD midguts at -4 h RPF. This revealed a significant reduction of both vesicular compartments and colocalisation between autophagic and lysosomal compartments was also reduced in *eff* KD midgut cells (Fig. 6c).

Together with our mosaic clone and *Atg1* morphology data, this suggests that Eff may be required to prevent premature midgut degradation in a non-cell-autonomous manner by slowing the turnover of autophagic vesicles.

### Cullin-4

**Fig. 7:**
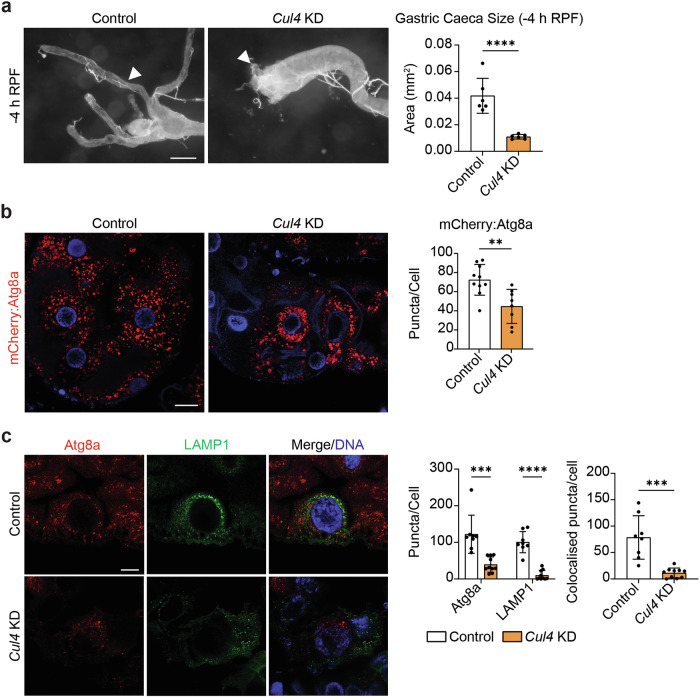
*Cul4* KD accelerates midgut degradation. **a** *Cul4* KD (*Mex-GAL4*/*+; pmCherry-Atg8a/UAS-Cul4i*) drastically upregulates ADCD and gastric caeca degradation compared to controls (*Mex-GAL4*/*w*^*1118*^*; pmCherry-Atg8a/+*). Quantitation of gastric caeca length at -4 h RPF represented as area ± SD (unpaired *t*-test, *n* = 6). **b** *Cul4* KD (*Mex-GAL4*/*+; pmCherry-Atg8a/UAS-Cul4i*) midguts have less Atg8a-positive vesicles (red) compared to controls (*Mex-GAL4*/*w*^*1118*^*; pmCherry-Atg8a/+*) at -4 h RPF. DNA stained by Hoechst (Blue). Quantitation of the number of Atg8a-positive vesicles represented as puncta/cell ± SD (unpaired *t*-test, *n* ≥ 8). **c** Immunostain for Atg8a (red) and LAMP1 (green) revealed a significant depletion of Atg8a and LAMP1 compartments upon *Cul4* KD (*Mex-GAL4* > *UAS-GFP-LAMP1*/*+; Cul4i/+*) compared to controls (*Mex-GAL4* > *UAS-GFP-LAMP1*/*w*^*1118*^) at -4 h RPF. DNA stained by Hoechst (Blue). Quantitation of Atg8a-positive and LAMP1-positive puncta represented as puncta/cell ± SD (unpaired t-test, *n* ≥ 8). Quantitation of overlap between Atg8a-positive compartment and LAMP1-positive compartment represented as colocalised puncta/cell ± SD (unpaired *t*-test, *n* ≥ 8). Scale bars = 125 µm (**a**), 20 µm (**b**) and 10 µm (**c**).

The E3 ligase, Cul4, has previously been described as an upstream regulator of autophagy [24, 25]. In our genetic screens, we reported an increase in the number of Atg8a-positive vesicles in *Cul4* KD clones and increased clonal cell size. Interestingly, we found that midgut-wide RNAi-mediated knockdown of *Cul4* resulted in significantly shorter gastric caeca compared to control midguts at -4 h RPF (Fig. 7a). Similar to the *eff* KD midgut phenotype, this discrepancy between mosaic clone depletion and midgut-wide knockdown of *Cul4* may indicate a non-cell-autonomous function of Cul4 in midgut removal. We also observed a significant reduction in the number of Atg8a-positive vesicles upon global depletion of *Cul4* in the midgut which was consistent with immunostaining for Atg8a (Fig. 7b, c). Strikingly, the lysosomal compartment was also depleted as revealed by GFP-LAMP1 immunostaining (Fig. 7c). Expectedly, because of fewer autophagosomes and lysosomes, significantly fewer vesicles positive for both Atg8a and LAMP1 were reported (Fig. 7c). Together, our mosaic clone and *Atg1* eye phenotype observations suggest that Cul4 may have a non-cell-autonomous role in coordinating timely midgut removal by regulating autophagosome-lysosome interactions.

### Supernumerary limbs

**Fig. 8:**
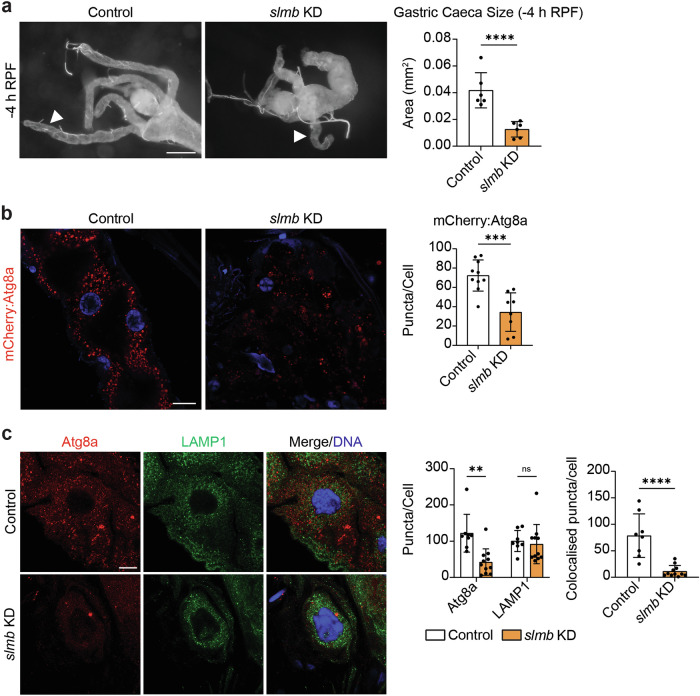
Slmb is required to prevent premature midgut degradation. **a** *slmb* KD (*Mex-GAL4*/*+; pmCherry-Atg8a/UAS-slmbi*) accelerates midgut appendage removal compared to controls (*Mex-GAL4*/*w*^*1118*^). Quantitation of gastric caeca length at -4 h RPF. Data presented as area ± SD (unpaired *t*-test, *n* = 6). **b** *slmb* KD (*Mex-GAL4*/+*; pmCherry-Atg8a/UAS-slmbi*) midguts have significantly less Atg8a-positive vesicles (red) compared to controls (*Mex-GAL4*/*w*^*1118*^*; pmCherry-Atg8a/+*) at -4 h RPF. DNA stained by Hoechst (Blue). Quantitation of the number of Atg8a-positive vesicles represented as puncta/cell ± SD (unpaired *t*-test, *n* ≥ 8). **c** Immunostain for Atg8a (red) and LAMP1 (green) shows a decrease in Atg8a-positive vesicles but no difference in LAMP1-positive vesicles in *slmb* KD (*Mex-GAL4* > *UAS-GFP-LAMP1*/ + *; UAS-slmbi/+*) midguts compared to controls (*Mex-GAL4* > *UAS-GFP-LAMP1*/*w*^*1118*^). DNA stained by Hoechst (Blue). Quantitation of Atg8a-positive and LAMP1-positive puncta represented as puncta/cell ± SD (unpaired t-test, *n* ≥ 8). Quantitation of overlap between Atg8a-positive compartment and LAMP1-positive compartment represented as colocalised puncta/cell ± SD (unpaired *t*-test, *n* ≥ 8). Scale bars = 125 µm (**a**), 20 µm (**b**) and 10 µm (**c**).

The roles of the E3 ligase, Slmb, and its mammalian ortholog, BTRC, have been described in autophagy initiation [26, 27]. Based on this evidence and together with our mosaic clone data, we hypothesised that Slmb may be an important regulator of midgut degradation. Midgut-wide RNAi-mediated knockdown of *slmb* resulted in drastically smaller and condensed midguts at -4 h RPF when compared to controls (Fig. 8a).

Next, we probed for Atg8a-positive vesicles in midguts with whole-tissue knockdown of *slmb* and observed a significant reduction in the number of Atg8a-positive vesicles at -4 h RPF that recapitulated the *slmb* KD mosaic clone phenotype (Fig. 8b). Immunostaining for Atg8a and GFP-LAMP1 confirmed a reduction in the number of Atg8a-positive vesicles upon *slmb* KD, however, no significant difference was observed within the LAMP1-positive compartment. In addition, Atg8a and LAMP1 double-positive staining was significantly reduced (Fig. 8c). Therefore, these data indicate a potential role for Slmb in negatively regulating autophagy-dependent midgut degradation by interacting with *Atg1* and controlling the number of autophagosomes in larval midgut cells.

## Discussion

Our RNAi screen for ubiquitination enzymes that regulate *Drosophila* autophagy-dependent midgut degradation has revealed 18 E1, E2 and E3 enzymes with potential roles in ADCD. Segregation of these enzymes based on their mosaic clone knockdown phenotype (i.e. Atg8a-positive vesicle number, Atg8a-positive vesicle size and cell size) offers valuable insights and scope for future studies into which stages of *Drosophila* midgut ADCD they may operate. To validate the findings from our mosaic clone and *Atg1* eye morphology screen, we further investigated the functions of the E2 enzyme, Eff, and the E3 ligases, Cul4 and Slmb, by knocking down these candidates in the whole *Drosophila* midgut at the onset of midgut degradation at -4 h RPF. In all three cases, we observed significantly smaller gastric caeca and extensive midgut remodelling compared to control midguts, suggesting premature initiation of midgut degradation prior to -4 h RPF. Depletion of *eff* and *slmb* resulted in dramatic reductions in the number of Atg8a-positive vesicles, recapitulating their mosaic clone phenotypes. Conversely, we reported a decrease in the number of Atg8a-positive vesicles in *Cul4* KD midguts but an increase in mosaic clones. Additionally, *eff* KD midguts were significantly smaller than controls unlike in mosaic clones where no cell size difference was reported. These delineations observed with *eff* and *Cul4* depletions from their mosaic clone phenotype may be due to non-cell-autonomous effects that requires further investigation. Depletion of *eff* and *Cul4* also showed significant loss of LAMP1-positive lysosomes, suggesting potential roles for these enzymes in lysosomal homeostasis. As previous studies have demonstrated the importance of E2 and E3 enzymes in regulating clearance of damaged lysosomes, it is possible that Eff and Cul4 may function at the level of the lysosome to mediate *Drosophila* midgut ADCD [28]. Loss of all three enzymes reduced the number of Atg8a-positive vesicles that colocalised with LAMP1. As our validations have not investigated alterations to autophagy flux as a result of these genes being depleted, it remains unclear whether the reduction in Atg8a- and LAMP1-positive vesicles is due to increased activation of autophagy or reduced lysosomal turnover of autophagic substrates. Finally, depletion of *eff*, *Cul4* and *slmb* caused significant pigment loss when *Atg1* was overexpressed. High levels of autophagy in adult *Drosophila* eyes, induced by *Atg1* overexpression, has been shown to cause significant pigment loss and alterations to eye size [20]. As *eff*, *Cul4* and *slmb* exacerbated the *Atg1* eye phenotype when depleted, this may be indicative of further activation of autophagy when knocked down. This may also explain why *eff* KD, *Cul4* KD and *slmb* KD midguts are extensively remodelled at -4 h RPF and have fewer Atg8a-positive vesicles, as early activation of autophagy in the midgut is known to drive premature midgut degradation [8].

Evolutionary-conservation of autophagy suggests the potential for our candidates to regulate *Drosophila* ADCD in a similar manner to the autophagy-related functions of their orthologs in other model organisms [29]. For example, the yeast ortholog of Eff, Ubc4, is involved in autophagy of proteasomes by interacting with the E3 ligases, San1, Rsp5 and Hul5 [30]. Similarly the mammalian ortholog, UBE2D2, ubiquitinates the selective autophagy receptor, Sequestosome-1 (SQSTM1/p62), under ubiquitin stress conditions, thereby activating autophagy [23]. UBE2D2 also has known roles in Parkin-mediated mitophagy [22]. As our studies demonstrate that loss of *eff* in the *Drosophila* midgut affects Atg8a- and LAMP1-positive compartments, future studies investigating which E3 ligases interact with Eff to regulate autophagy will aid in our understanding of how Eff can facilitate *Drosophila* midgut ADCD. Although a yeast homologue of Cul4 has not been defined, the mammalian ortholog, CUL4A, has notable roles in autophagy regulation. The CUL4A-DDB1-WDFY1 E3 complex ubiquitinates LAMP2 on damaged lysosomes to promote lysophagy [31]. CUL4A also forms a complex with AMBRA1 and DDB1 to ubiquitinate BECN1 for the induction of starvation-induced autophagy [32]. Additionally, CUL4A can control protein levels of AMBRA1, whereby association of CUL4A with AMBRA1 causes AMBRA1 degradation and termination of autophagy [33]. Interestingly, in *Aedes aegypti* (yellow fever mosquito), AMBRA1 deficiency prevented autophagy-dependent midgut degradation and resulted in pupal lethality [34]. However, as there is no ortholog of AMBRA1 in *Drosophila*, our study hints at a potential novel role for Cul4 in mediating *Drosophila* midgut degradation that should be further explored. Finally, the mammalian ortholog of Slmb, BTRC, has been shown to ubiquitinate ULK1 in breast cancer cells, leading to mitophagy deficiency and cancer cell metastasis [27]. As our study has demonstrated a genetic interaction between *slmb* and *Atg1* in the adult *Drosophila* eye, how Slmb interacts with *Atg1* in midgut cells to cause midgut degradation remains to be explored.

There are other candidate regulators in our genetic screen that we did not further validate but display considerable autophagic defects in mosaic clones. For example, loss of some candidates resulted in larger Atg8a-positive vesicle size that may be indicative of large autophagic bodies persisting in midgut cells during degradation. Indeed, multiple studies have found that defects in autophagy result in larger Atg8a-positive vesicles [35–37]. However, as we have not determined whether these vesicles are autophagosomes or autolysosomes, further investigations are required to deduce the stage of autophagy at which this defect occurs. Additionally, it is unknown how enlargement of Atg8a-positive vesicles upon knockdown of our candidate genes affects midgut remodelling. Finally, knockdown of *hyperplastic discs* (*hyd*) displayed no changes to the Atg8a compartment but resulted in larger clonal cells and demonstrated differences in eye morphology when *Atg1* was overexpressed (Supplementary Fig. 1). As midgut remodelling requires ecdysone-dependent downregulation of growth signalling and Dpp signalling, it is possible that the *hyd* mosaic clone phenotype may be the result of altered upstream signalling pathways that initiate autophagy-dependent midgut degradation [3, 5, 7, 9]. Indeed, the Hedgehog and Dpp signalling pathways are known to be negatively regulated by Hyd in the *Drosophila* eye and wing disc in a tissue-specific and spatiotemporal manner [38].

Overall, our RNAi-mediated genetic screen has identified several ubiquitination enzymes that have putative roles in driving *Drosophila* midgut ADCD. Mechanistic insights into how these enzymes regulate midgut degradation will increase our understanding of the interplay between the ubiquitination system and autophagic machinery, and how this process results in *Drosophila* midgut ADCD.

## Materials and methods

### Drosophila transgenic lines and maintenance

All *Drosophila* transgenic lines (serial number and source) utilised in this study are provided in Supplementary Table 1. All flies were maintained at 18^o^C and experimental crosses were performed at 25^o^C on *Drosophila* media [18.75% compressed yeast, 10% treacle, 10% polenta, 2.5% tegosept (10% parahydroxybenzoate in ethanol), 1.5% acid mix (47% proionic acid, 4.7% orthophosphoric acid) and 1% w/v agar].

### Larval staging and midgut morphology analyses

Larvae were raised on *Drosophila* media supplemented with 0.05% bromophenol blue (Sigma-Aldrich, B6131). Wandering third-instar larvae were collected and placed on moist Whatmann paper. Gut clearance was visualised by the reduction of the bromophenol blue dye from the gut [39, 40]. Appropriately-staged animals were dissected in 1xPBS and midguts were fixed in 4% PFA before imaging on a stereozoom microscope (Olympus, Tokyo, Japan). Gastric caeca size was measured on Photoshop (Adobe, San Jose, CA, USA) using the magnetic lasso tool and histogram function to determine area in pixels. To convert values to biological measurements, pixel values were multiplied by the pixel dimension area of images.

### Eye morphology analysis

Adult male flies from crosses performed at 25^o^C were collected 1 day post-eclosion and frozen at -20^o^C for up to 1 day prior to imaging. Flies were mounted with nail varnish and eyes were imaged on a stereozoom microscope (Olympus, Tokyo, Japan) using 4x optical zoom. Eye size and aberrant pigment area was measured using the freehand selection tool and “measure” function and biological scale measurements were established on ImageJ (Bethesda, MD, USA) by imaging a haemocytometer grid under 4x optical zoom.

### Live imaging of Drosophila midguts

For mCherry-Atg8a imaging, appropriately-staged animals were dissected in 1xPBS with Hoechst 33342 (2 μg/ml, Sigma-Aldrich) and midguts were imaged immediately using a Carl Zeiss LSM 800 Axio Observer 7 laser scanning confocal microscope (Carl Zeiss Microscopy, Jena, Germany). Quantitation of puncta was performed using ImageJ (Bethesda, MD, USA). Puncta size > 10 pixels were quantitated.

### Immunostaining of Drosophila midguts

Appropriately-staged animals were dissected in 1xPBS and larval midguts were fixed in 4% v/v paraformaldehyde at room temperature for 45 min. Midguts were washed 3 × 5 min in 1xPBTw (PBS + 0.1% Tween-20) and 1 × 10 min in 1xPBTx (PBS + 0.1% Triton-X) before blocking in 1xPBTx + 1% BSA for 1 h at room temperature. Samples were incubated overnight at 4^o^C with primary antibodies diluted in 1xPBTx. Primary antibodies used were rabbit anti-GABARAP 1:200 (Abcam ab109364) and goat anti-GFP 1:500 (Rockford 600-101-215). Midguts were washed the following day in 4 × 30 min 1xPBTx and incubated in secondary antibodies for 1 h at room temperature. Secondary antibodies used were anti-rabbit Alexa-FLUOR 555 and anti-goat Alexa-FLUOR 488 (1:200, Molecular Probes, Eugene, CA, USA). Samples were washed 3 × 10 min in 1xPBTx and stained with Hoechst 33342 (2 µg/µL) for 1 min. Samples were washed 3 × 10 min in 1xPBTx and mounted in ProLong® Gold Antifade (Thermo Scientific). Imaging was performed on a Zeiss LSM 800 confocal microscope (Carl Zeiss Microscopy, Jena, Germany). ImageJ (Bethesda, MD, USA) was used to quantitate Atg8a- and LAMP1-positive puncta with size >0.1 µm^2^. ImageJ (Bethesda, MD, USA) was used to determine colocalisation between puncta. Colour images were split into individual binary images and thresholded. The “image calculator” and “AND” function was utilised on Atg8a- and LAMP1-positive channels which were set as Image A and Image B, respectively, to derive a new binary image of double-positive puncta. These were quantitated using the “analyse particles” function by setting the size threshold to > 0.1 µm^2^.

### Quantitative real time polymerase chain reaction (qRT-PCR)

To validate genetic knockdown efficiency, whole adult flies were collected within 1 day of eclosion, frozen at -20^o^C and homogenised in TRIzol® reagent (Life Technologies, Carlsbad, CA) according to the manufacturer’s protocol. High Capacity cDNA Reverse Transcription Kit (Applied Biosciences) was utilised to create cDNA from 1 μg of RNA, RNaseOUT™ Recombinant Ribonuclease Inhibitor (Thermo Fisher) and random primers, according to the manufacturer’s protocol. KAPA SYBR® FAST was used to perform qRT-PCR on a Rotor-Gene Q (Qiagen, Valencia, CA, USA) with the operating software, Rotor-Gene, according to the manufacturer’s protocol. Each reaction was performed in triplicate with three flies per sample. RNA expression was normalised to the housekeeping gene, *rp49* (beta-actin in humans) using Rotor-Gene Q 2.3.4 (Build 3) software. Primers utilised for qRT-PCR and RNAi line validations are provided in Nicolson [17].

### Confocal microscopy

Confocal images were obtained on a Carl Zeiss LSM 800 Axio Observer 7 laser scanning confocal microscope with 405 nm (5 mW), 488 nm (10 mW), 561 nm (10 mW) and 640 nm (5 mW) lasers. Objectives utilised were the PlanApo 40 x /1.3 or 63 x /1.4 Oil DIC objectives (Carl Zeiss Microscopy, Jena, Germany). Zen 2011 (Black Edition) software was utilised to capture images at 40x and Airyscan detector was used to capture images at 63x [41]. Zen 3.4 (Blue Edition) was used to batch process images.

### Statistical analyses

All statistical analyses were performed using GraphPad Prism version 10.0.0 for Windows, GraphPad Software (Boston, Massachusetts USA). Mosaic clones were analysed by a paired t-test. Eye morphology data were analysed by Two-way ANOVA (Uncorrected Fisher’s LSD test). All other data sets were analysed by an unpaired t-test with ns = non-significant, *p < 0.05, **p < 0.01, ***p < 0.001 and ****p < 0.0001.

## Supplementary information

Supplemental Material

## Data Availability

The data generated in this study are available within the article and its supplementary data files. All raw data are available upon request.
