# Supplementary material for: Identification of new candidates regulating autophagy-dependent midgut degradation in Drosophila melanogaster
Source: Cell Death Discov. 2025 Apr 16;11:181. doi: 10.1038/s41420-025-02474-0 (PMC12003636; doi:10.1038/s41420-025-02474-0)
Supplement: Supplementary file 1 — Supplemental Material [file 41420_2025_2474_MOESM1_ESM.docx]

| **Gene** | **Drosophila transgenic line** | **Stock Number** | **Source** |
| --- | --- | --- | --- |
| GMR-GAL4 | w[*]; P{w[+mC]=GAL4-ninaE.GMR}12 | 1104 | Bloomington Drosophila Stock Centre (Indiana University, USA) |
|  | GMR-GAL4/CyOGFP; Atg1-GFP/TM2 |  | In-house |
|  | hsFLP; pmCherry-Atg8a; Act > CD2 > GAL4, UAS-nlsGFP/TM6B |  | Eric Baehrecke (UMass Chan Medical School, USA) |
|  | mex-GAL4 |  | Richard Burke (Monash University, Australia) |
|  | mex-GAL4/CyOactGFP; pmCherryAtg8a/TM6BTS |  | In-house |
|  | mex-GAL4, UAS-LAMP1-GFP/CyO actGFP; +/TM6B or TM2 |  | Helena Richardson (La Trobe University, Australia) |
| Cul4 | UAS-Cul4 RNAi |  | Kieran Harvey (Peter MacCallum Cancer Centre, Monash University, Australia) |
| dor | UAS-dor RNAi |  | Kieran Harvey (Peter MacCallum Cancer Centre, Monash University, Australia) |
| eff^35431^ | y[1] sc[*] v[1] sev[21]; P{y[+t7.7] v[+t1.8]=TRiP.GL00355}attP2 | 35431 | Bloomington Drosophila Stock Centre (Indiana University, USA) |
| eff^R2^ | UAS-eff RNAi | 7425R-2 | Kieran Harvey (Peter MacCallum Cancer Centre, Monash University, Australia) |
| hyd | UAS-hyd RNAi |  | Kieran Harvey (Peter MacCallum Cancer Centre, Monash University, Australia) |
| lt | UAS-lt RNAi |  | Kieran Harvey (Peter MacCallum Cancer Centre, Monash University, Australia) |
| LUBEL | UAS-LUBEL RNAi (KK) | 100651 | Vienna Drosophila Resource Centre (Vienna) |
| Mi-2 | y[1] v[1]; P{y[+t7.7] v[+t1.8]=TRiP.HMC03329}attP40 | 51774 | Bloomington Drosophila Stock Centre (Indiana University, USA) |
| mr | y[1] v[1]; P{y[+t7.7] v[+t1.8]=TRiP.HMS02023}attP40 | 40856 | Bloomington Drosophila Stock Centre (Indiana University, USA) |
| ntc | UAS-ntc RNAi |  | Kieran Harvey (Peter MacCallum Cancer Centre, Monash University, Australia) |
| Pex2 | UAS-Pex2 RNAi |  | Kieran Harvey (Peter MacCallum Cancer Centre, Monash University, Australia) |
| Psc | y[1] sc[*] v[1] sev[21]; P{y[+t7.7] v[+t1.8]=TRiP.HMS01706}attP40 | 38261 | Bloomington Drosophila Stock Centre (Indiana University, USA) |
| Prp19 | UAS-Prp19 RNAi/CyO |  | Kieran Harvey (Peter MacCallum Cancer Centre, Monash University, Australia) |
| Rnf11 | y[1] v[1]; P{y[+t7.7] v[+t1.8]=TRiP.HMJ22085}attP40 | 58136 | Bloomington Drosophila Stock Centre (Indiana University, USA) |
| sip3 | y[1] v[1]; P{y[+t7.7] v[+t1.8]=TRiP.HMJ23236}attP40 | 61344 | Bloomington Drosophila Stock Centre (Indiana University, USA) |
| slmb | y[1] sc[*] v[1] sev[21]; P{y[+t7.7] v[+t1.8]=TRiP.HMS00946}attP2 | 33986 | Bloomington Drosophila Stock Centre (Indiana University, USA) |
| Uba1 | y[1] v[1]; P{y[+t7.7] v[+t1.8]=TRiP.JF01977}attP2 | 25957 | Bloomington Drosophila Stock Centre (Indiana University, USA) |
| Ube4a | UAS-Ube4a RNAi (GD) | 31413 | Vienna Drosophila Resource Centre (Vienna) |
| w1118 | w[1118] | 3605 | Bloomington Drosophila Stock Centre (Indiana University, USA) |

**Supplementary Table 1:** List of *Drosophila* transgenic lines utilised in this study. Serial numbers and sources provided where appropriate.

| ***Drosophila* *melanogaster***  **(fruit fly)** | ***Saccharomyces cerevisiae***  **(yeast)** | ***Homo sapiens***  **(human)** |
| --- | --- | --- |
| *Cul4* | *-* | *Cul4a* |
| *dor* | *Pep3* | *Vps18* |
| *eff* | *Ubc4* | *Ube2d2* |
| *hyd* | *-* | *Ubr5* |
| *lt* | *Vps41* | *Vps41* |
| *LUBEL* | *Itt1* | *Rnf31* |
| *Mi-2* | *Irc5* | *Chd5* |
| *mr* | *Apc2* | *Anapc2* |
| *ntc* | *-* | *-* |
| *Pex2* | *Pex2* | *Pex2* |
| *Prp19* | *Prp19* | *Prpf19* |
| *Psc* | *-* | *Bmi1* |
| *Rchy1* | *-* | *Rchy1* |
| *Rnf11* | *-* | *Rnf11* |
| *sip3* | *Hrd1* | *Syvn1* |
| *slmb* | *-* | *Btrc* |
| *Uba1* | *Uba1* | *Uba1* |
| *Ube4a* | *Ufd2* | *Ube4a* |

**Supplementary Table 2:** List of candidate regulators of autophagy-dependent midgut degradation and their conserved orthologs in yeast and humans.


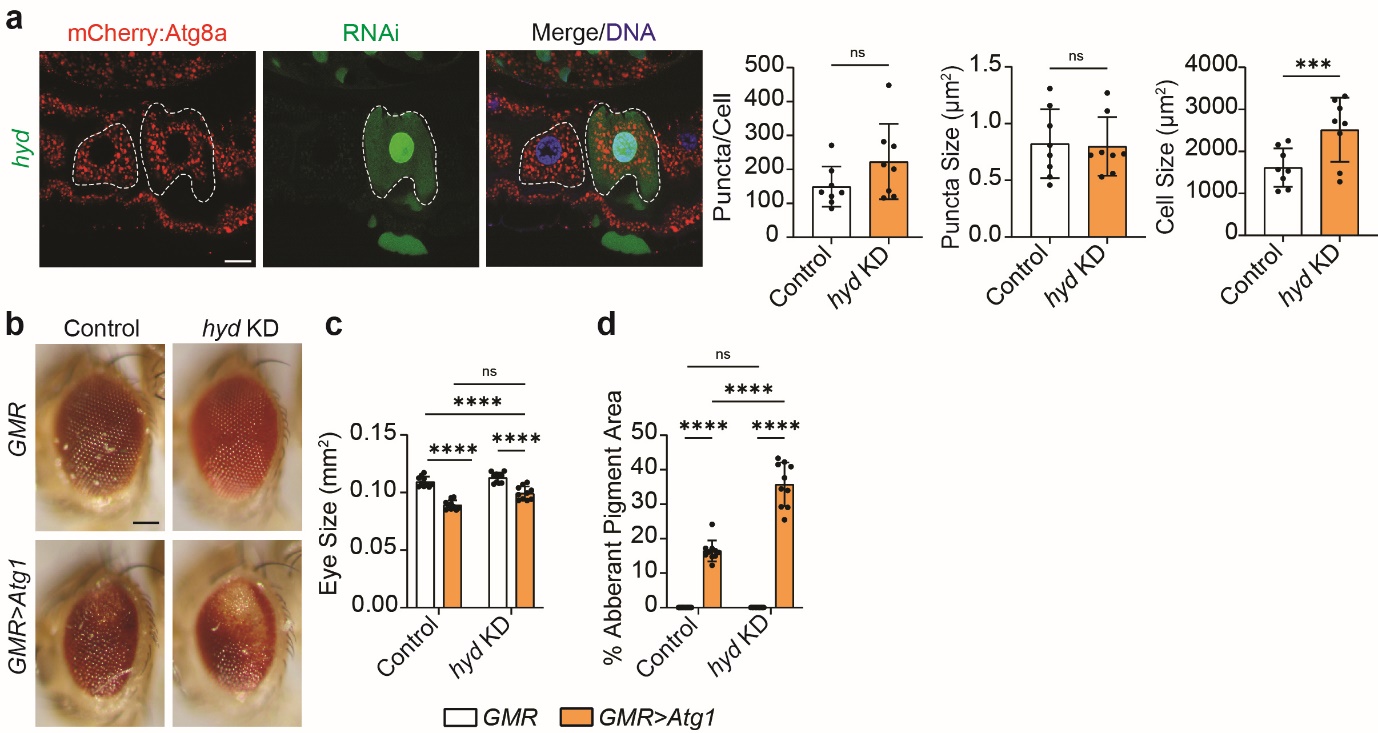


**Supplementary Figure 1: Mosaic clone mutants that cause an increase in cell size. a** *hyd* KD (*hsFLP; pmCherry-Atg8a/+; Act* > *CD2* > *GAL4, UAS-nlsGFP/UAS-hydi*) in GFP-labelled midgut cells compared to non-GFP control cells (white dashed lines indicate cell boundaries) at -4 h RPF. Quantitation of Atg8a puncta per cell represented as puncta/cell ± SD (paired t-test, *n* = 8). Quantitation of Atg8a puncta size represented as µm^2^ ± SD (paired t-test, *n* = 8). Quantitation of cell size represented as µm^2^ ± SD (paired t-test, *n* = 8). **b** Representative eye phenotypes for *hyd* KD under *GMR-GAL4* only (*GMR-GAL4/+; UAS-hydi/+*) and *Atg1* overexpression conditions (*GMR-GAL4/+; GMR>Atg1/UAS-hydi*). **c** Quantitation of eye size represented as mm^2^ ± SD (Two-way ANOVA with Uncorrected Fisher’s LSD test, *n* ≥ 10). **d** Quantitation of aberrant pigment area represented as % Aberrant Pigment Area ± SD (Two-way ANOVA with Uncorrected Fisher’s LSD test, *n* ≥ 10). Scale bar = 20μm (**a**) and 125μm (**b**).
